# Supplementary material for: Machine Learning-Based Classification of Transcriptome Signatures of Non-Ulcerative Bladder Pain Syndrome
Source: Int J Mol Sci. 2024 Jan 26;25(3):1568. doi: 10.3390/ijms25031568 (PMC10855300; doi:10.3390/ijms25031568)
Supplement: Supplementary file 1 [file ijms-25-01568-s001.zip › ijms-2811523-supplementary.pdf]

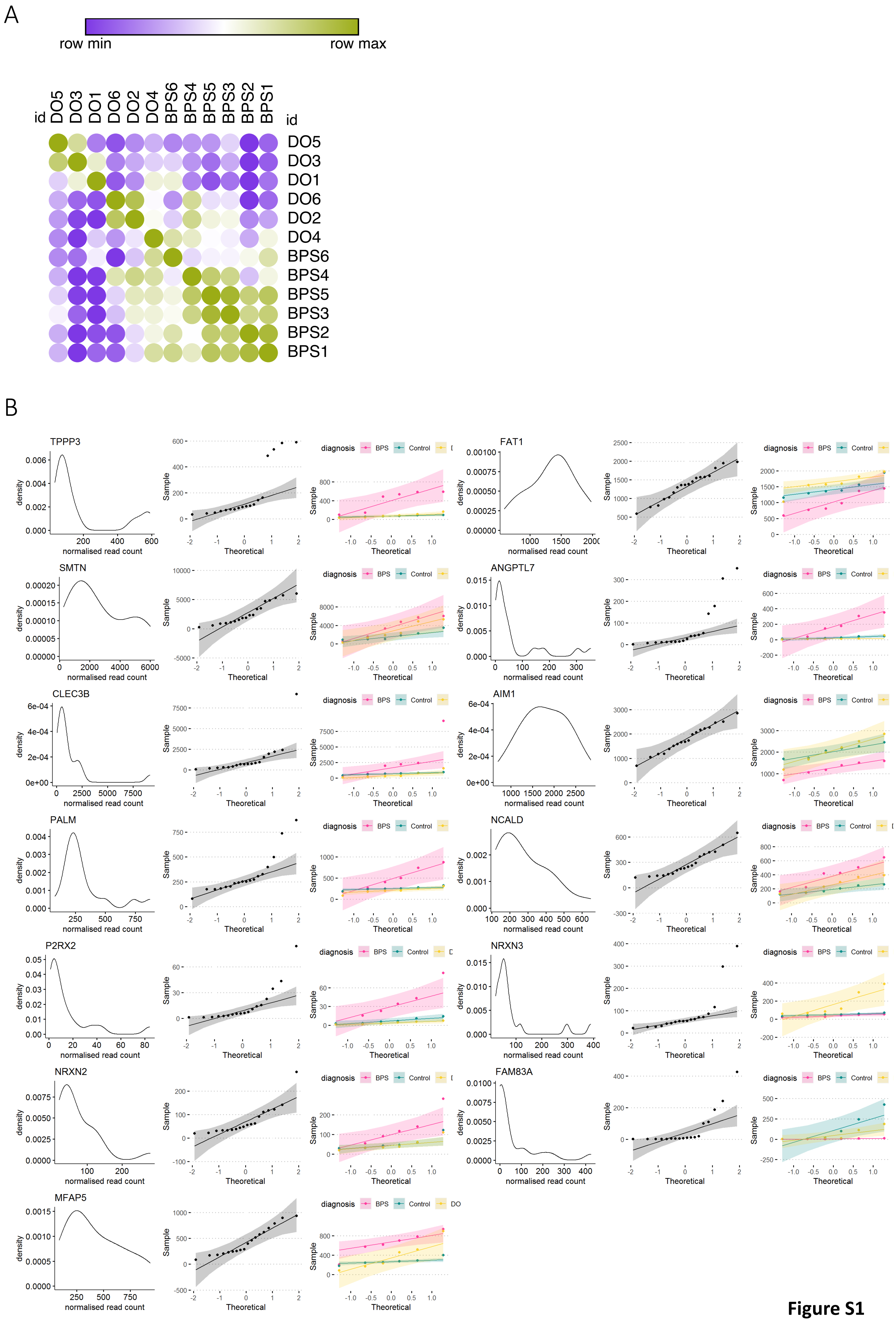

A
row min
row max
id
DO5
DO3
DO1
DO6
DO2
DO4
BPS6
BPS4
BPS5
BPS3
BPS2
BPS1
B
TPPP3
FAT1
SMTN
ANGPTL7
CLEC3B
AIM1
PALM
NCALD
P2RX2
NRXN3
NRXN2
FAM83A
MFAP5
density
normalised read count
Sample
Theoretical
diagnosis
BPS
Control
DO

Figure S1

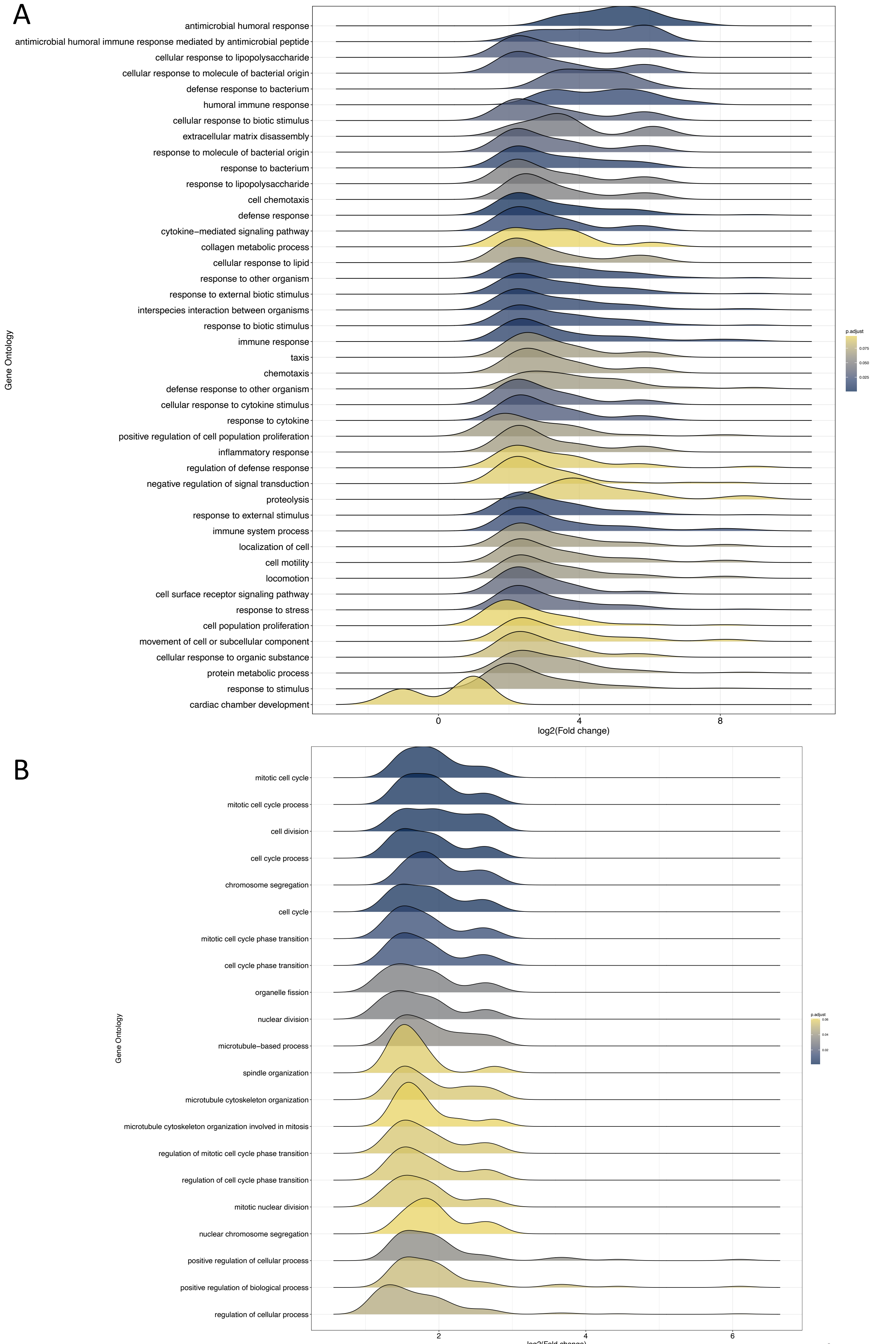
A
antimicrobial humoral response
antimicrobial humoral immune response mediated by antimicrobial peptide
cellular response to lipopolysaccharide
cellular response to molecule of bacterial origin
defense response to bacterium
humoral immune response
cellular response to biotic stimulus
extracellular matrix disassembly
response to molecule of bacterial origin
response to bacterium
response to lipopolysaccharide
cell chemotaxis
defense response
cytokine−mediated signaling pathway
collagen metabolic process
cellular response to lipid
response to other organism
response to external biotic stimulus
interspecies interaction between organisms
response to biotic stimulus
immune response
taxis
chemotaxis
defense response to other organism
cellular response to cytokine stimulus
response to cytokine
positive regulation of cell population proliferation
inflammatory response
regulation of defense response
negative regulation of signal transduction
proteolysis
response to external stimulus
immune system process
localization of cell
cell motility
locomotion
cell surface receptor signaling pathway
response to stress
cell population proliferation
movement of cell or subcellular component
cellular response to organic substance
protein metabolic process
response to stimulus
cardiac chamber development
Gene Ontology
0
4
8
log2(Fold change)
p.adjust
0.075
0.050
0.025
B
mitotic cell cycle
mitotic cell cycle process
cell division
cell cycle process
chromosome segregation
cell cycle
mitotic cell cycle phase transition
cell cycle phase transition
organelle fission
nuclear division
microtubule−based process
spindle organization
microtubule cytoskeleton organization
microtubule cytoskeleton organization involved in mitosis
regulation of mitotic cell cycle phase transition
regulation of cell cycle phase transition
mitotic nuclear division
nuclear chromosome segregation
positive regulation of cellular process
positive regulation of biological process
regulation of cellular process
Gene Ontology
2
4
6
log2(Fold change)
p.adjust
0.06
0.04
0.02

Figure S2

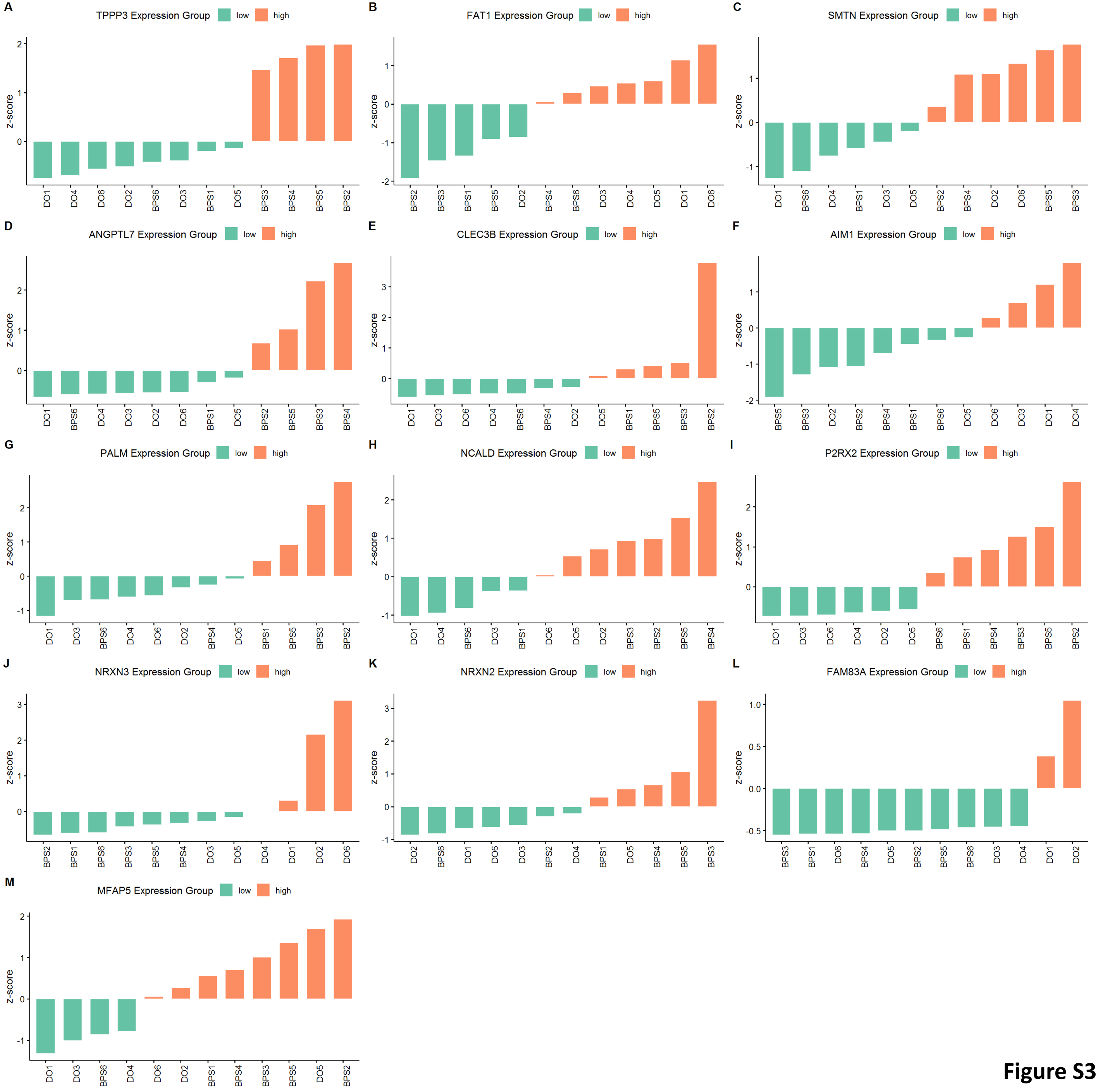
A
TPPP3 Expression Group low high
z-score
DO1 DO4 DO6 DO2 BPS6 DO3 BPS1 DO5 BPS3 BPS4 BPS5 BPS2
B
FAT1 Expression Group low high
z-score
BPS2 BPS3 BPS1 BPS5 DO2 BPS4 BPS6 DO3 DO4 DO5 DO1 DO6
C
SMTN Expression Group low high
z-score
DO1 BPS6 DO4 BPS1 DO3 DO5 BPS2 BPS4 DO2 DO6 BPS5 BPS3
D
ANGPTL7 Expression Group low high
z-score
DO1 BPS6 DO4 DO3 DO2 DO6 BPS1 DO5 BPS2 BPS5 BPS3 BPS4
E
CLEC3B Expression Group low high
z-score
DO1 DO3 DO6 DO4 BPS6 BPS4 DO2 DO5 BPS1 BPS5 BPS3 BPS2
F
AIM1 Expression Group low high
z-score
BPS5 BPS3 DO2 BPS2 BPS4 BPS1 BPS6 DO5 DO6 DO3 DO1 DO4
G
PALM Expression Group low high
z-score
DO1 DO3 BPS6 DO4 DO6 DO2 BPS4 DO5 BPS1 BPS5 BPS3 BPS2
H
NCALD Expression Group low high
z-score
DO1 DO4 BPS6 DO3 BPS1 DO6 DO5 DO2 BPS3 BPS2 BPS5 BPS4
I
P2RX2 Expression Group low high
z-score
DO1 DO3 DO6 DO4 DO2 DO5 BPS6 BPS1 BPS4 BPS3 BPS5 BPS2
J
NRXN3 Expression Group low high
z-score
BPS2 BPS1 BPS6 BPS3 BPS5 BPS4 DO3 DO5 DO4 DO1 DO2 DO6
K
NRXN2 Expression Group low high
z-score
DO2 BPS6 DO1 DO6 DO3 BPS2 DO4 BPS1 DO5 BPS4 BPS5 BPS3
L
FAM83A Expression Group low high
z-score
BPS3 BPS1 DO6 BPS4 DO5 BPS2 BPS5 BPS6 DO3 DO4 DO1 DO2
M
MFAP5 Expression Group low high
z-score
DO1 DO3 BPS6 DO4 DO6 DO2 BPS1 BPS4 BPS3 BPS5 DO5 BPS2

Figure S3

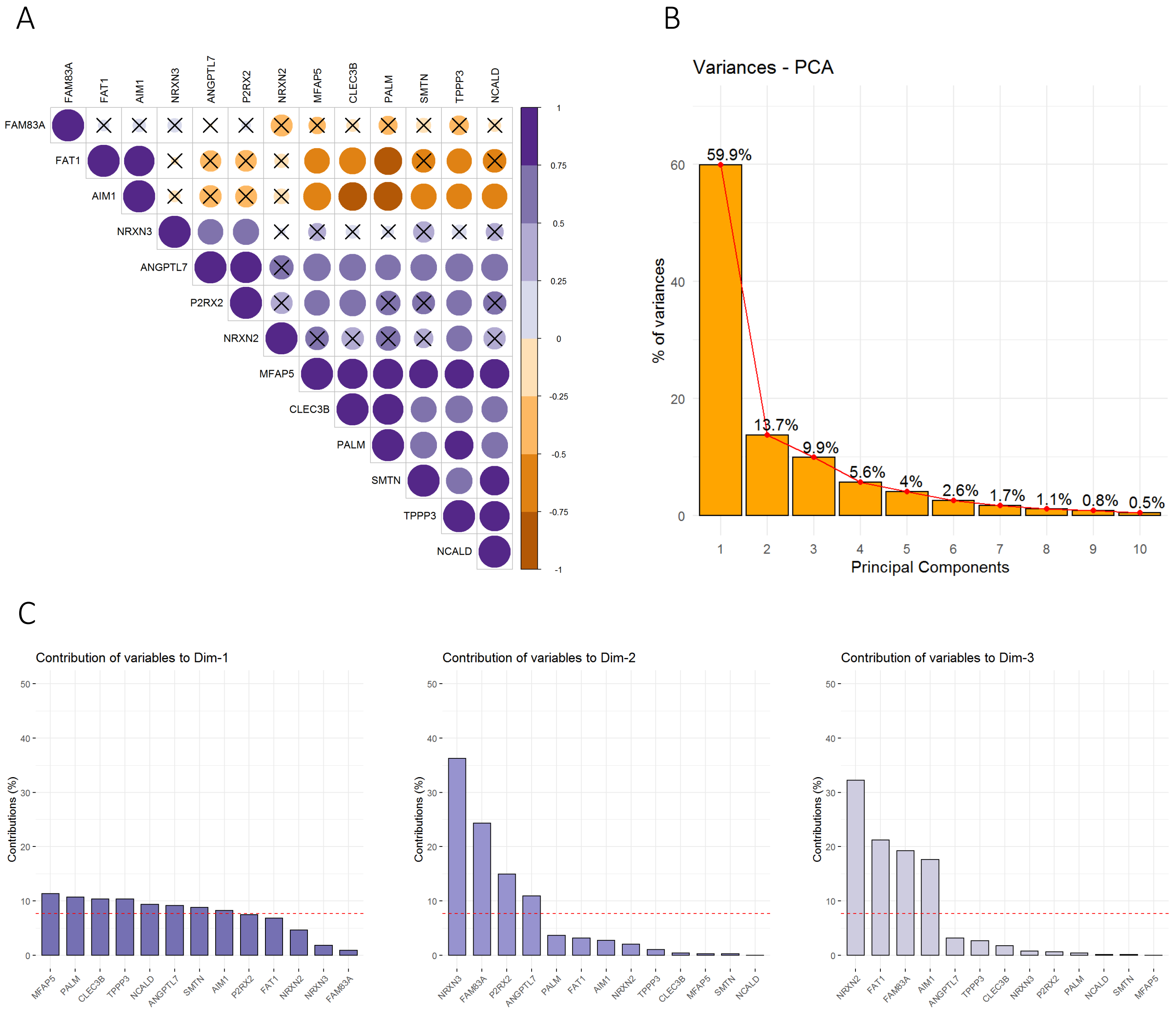
A
FAM83A
FAT1
AIM1
NRXN3
ANGPTL7
P2RX2
NRXN2
MFAP5
CLEC3B
PALM
SMTN
TPPP3
NCALD
B
Variances - PCA
59.9%
13.7%
9.9%
5.6%
4%
2.6%
1.7%
1.1%
0.8%
0.5%
% of variances
Principal Components
C
Contribution of variables to Dim-1
Contribution of variables to Dim-2
Contribution of variables to Dim-3
Contributions (%)

Figure S4

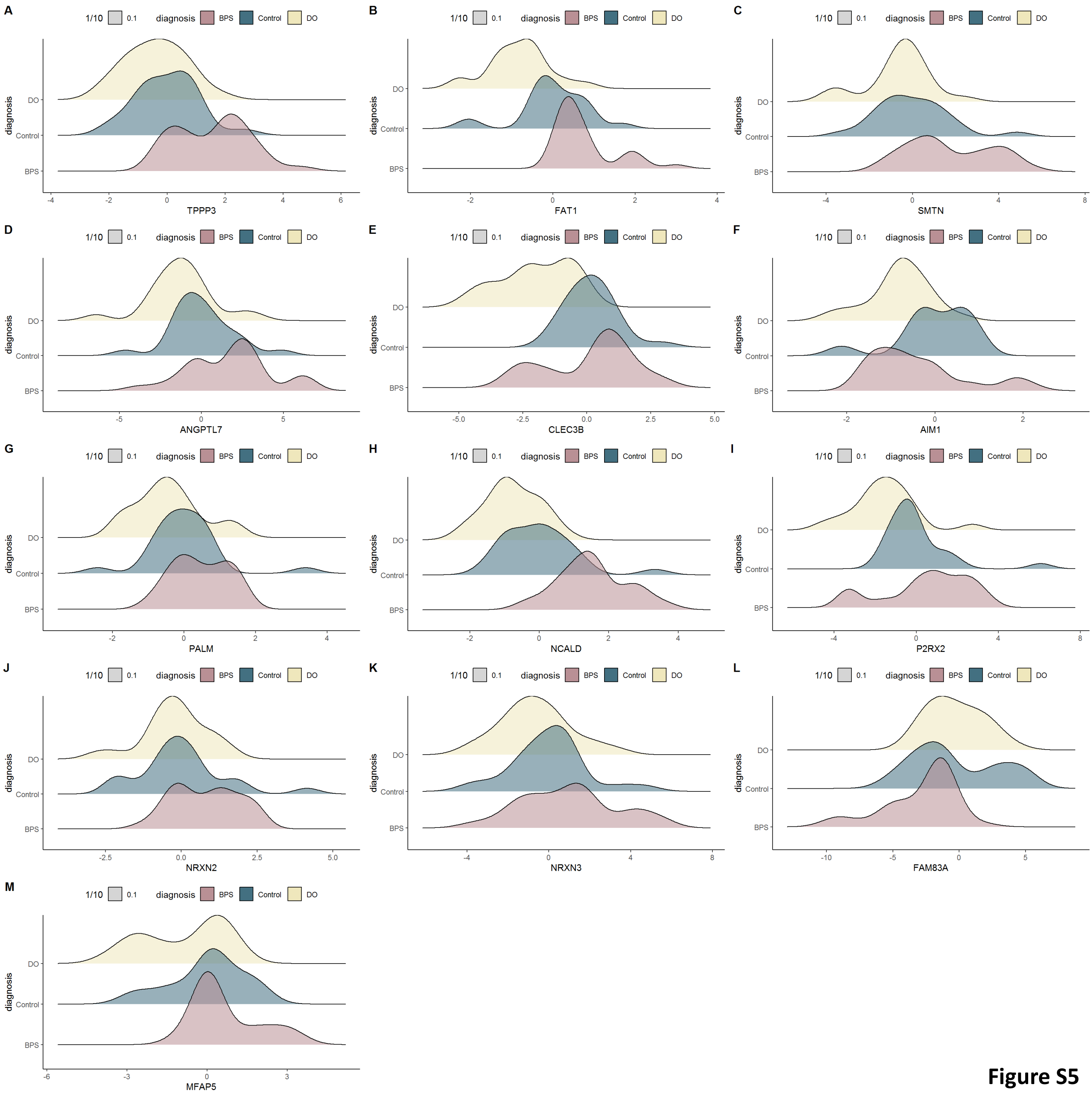
A
1/10
0.1
diagnosis
BPS
Control
DO
TPPP3
B
FAT1
C
SMTN
D
ANGPTL7
E
CLEC3B
F
AIM1
G
PALM
H
NCALD
I
P2RX2
J
NRXN2
K
NRXN3
L
FAM83A
M
MFAP5

Figure S5

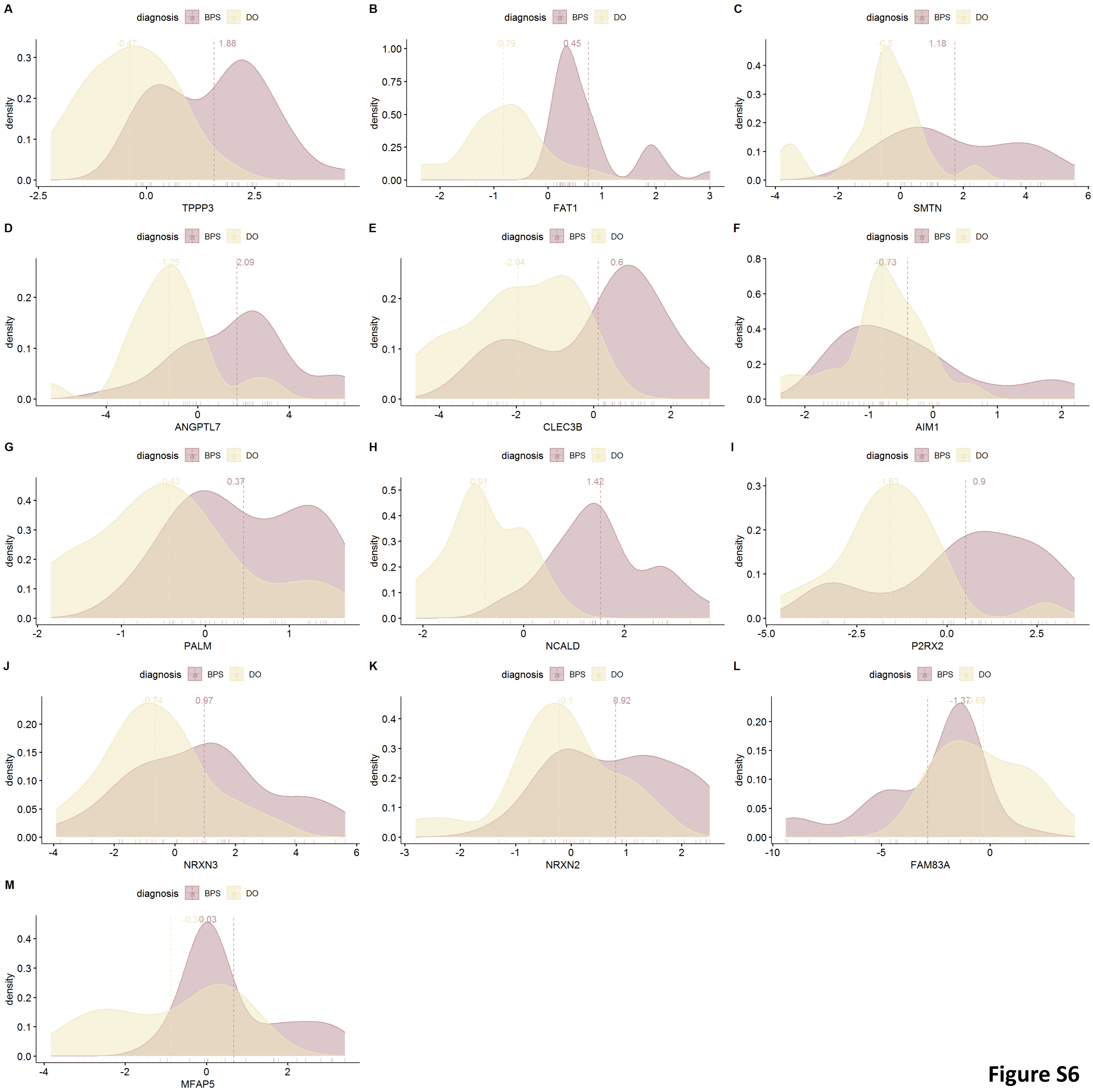
A
B
C
D
E
F
G
H
I
J
K
L
M
diagnosis
BPS
DO
density
TPPP3
FAT1
SMTN
ANGPTL7
CLEC3B
AIM1
PALM
NCALD
P2RX2
NRXN3
NRXN2
FAM83A
MFAP5
-0.47
1.88
-0.79
0.45
-0.5
1.18
-1.25
2.09
-2.04
0.6
-0.73
-0.43
0.37
-0.91
1.42
-1.63
0.9
-0.74
0.97
-0.1
0.92
-1.37
0.69
0.03

Figure S6

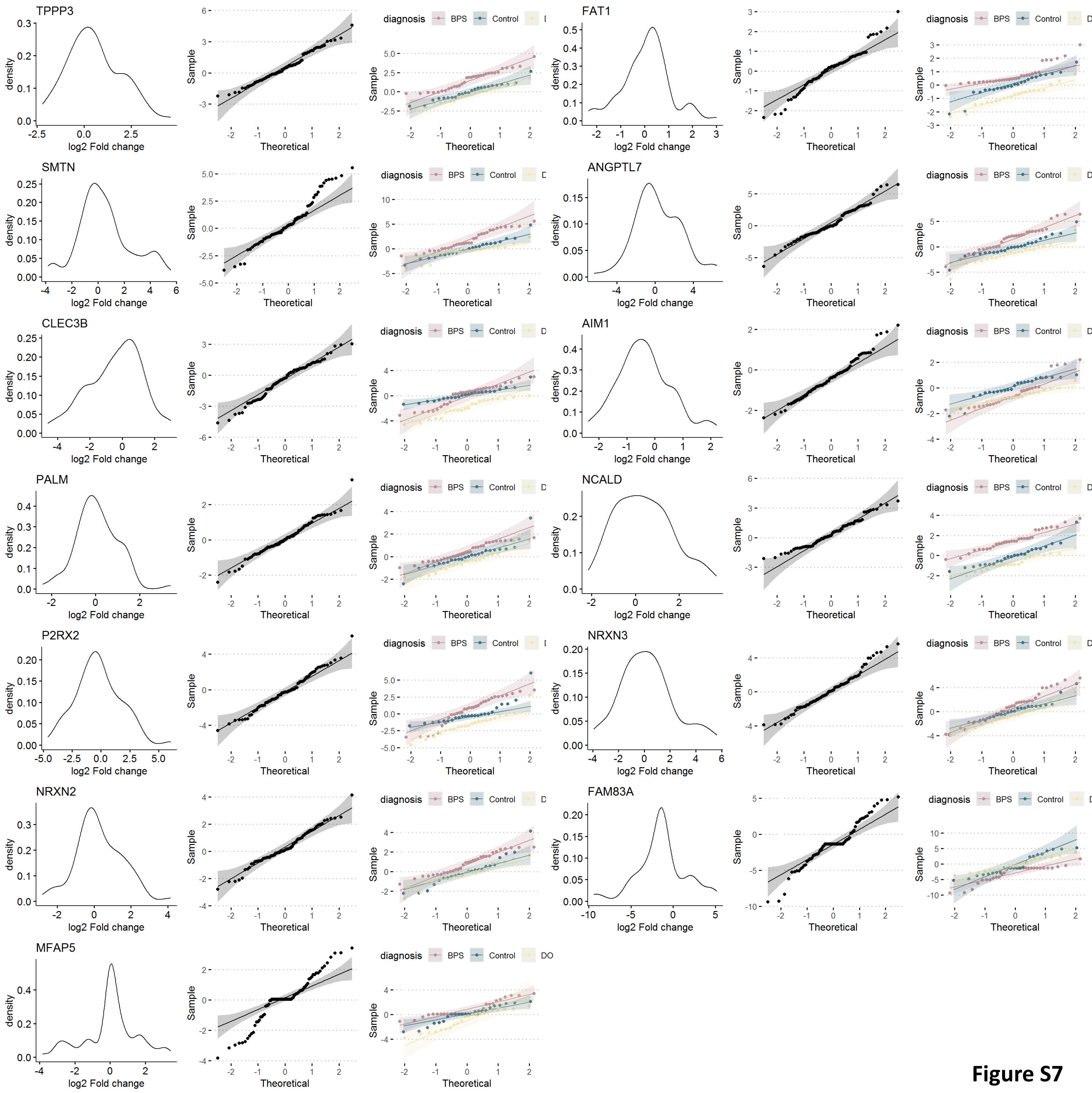
TPPP3
FAT1
SMTN
ANGPTL7
CLEC3B
AIM1
PALM
NCALD
P2RX2
NRXN3
NRXN2
FAM83A
MFAP5
density
log2 Fold change
Sample
Theoretical
diagnosis
BPS
Control
DO

Figure S7

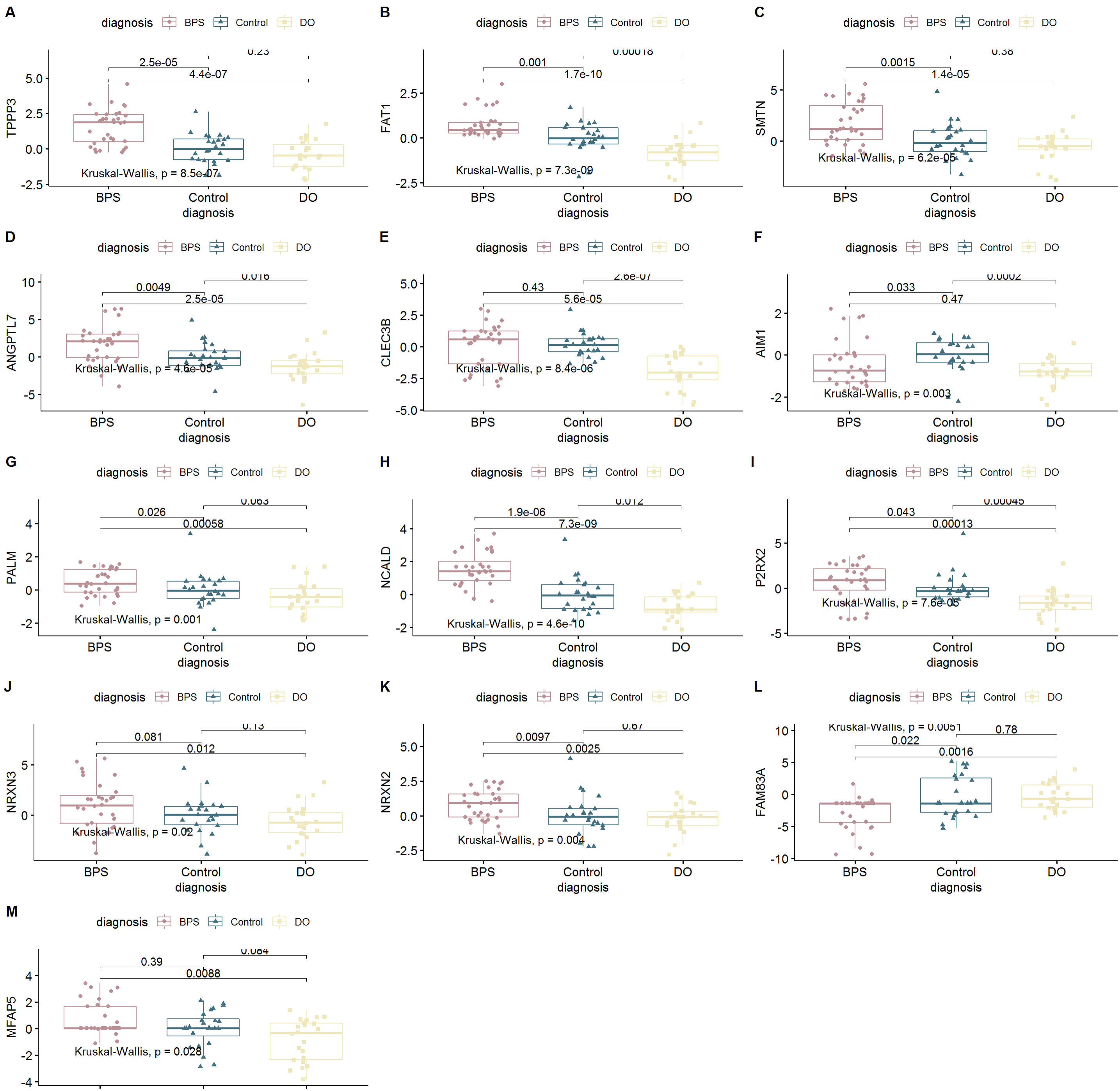
A
diagnosis BPS Control DO
TPPP3
2.5e-05
0.23
4.4e-07
Kruskal-Wallis, p = 8.5e-07
BPS Control DO
diagnosis
B
FAT1
0.001
0.00018
1.7e-10
Kruskal-Wallis, p = 7.3e-09
C
SMTN
0.0015
0.38
1.4e-05
Kruskal-Wallis, p = 6.2e-05
D
ANGPTL7
0.0049
0.016
2.5e-05
Kruskal-Wallis, p = 4.6e-05
E
CLEC3B
0.43
2.6e-07
5.6e-05
Kruskal-Wallis, p = 8.4e-06
F
AIM1
0.033
0.0002
0.47
Kruskal-Wallis, p = 0.003
G
PALM
0.026
0.063
0.00058
Kruskal-Wallis, p = 0.001
H
NCALD
1.9e-06
0.012
7.3e-09
Kruskal-Wallis, p = 4.6e-10
I
P2RX2
0.043
0.00045
0.00013
Kruskal-Wallis, p = 7.6e-05
J
NRXN3
0.081
0.13
0.012
Kruskal-Wallis, p = 0.02
K
NRXN2
0.0097
0.67
0.0025
Kruskal-Wallis, p = 0.004
L
FAM83A
Kruskal-Wallis, p = 0.0051
0.022
0.78
0.0016
M
MFAP5
0.39
0.084
0.0088
Kruskal-Wallis, p = 0.028

Figure S8

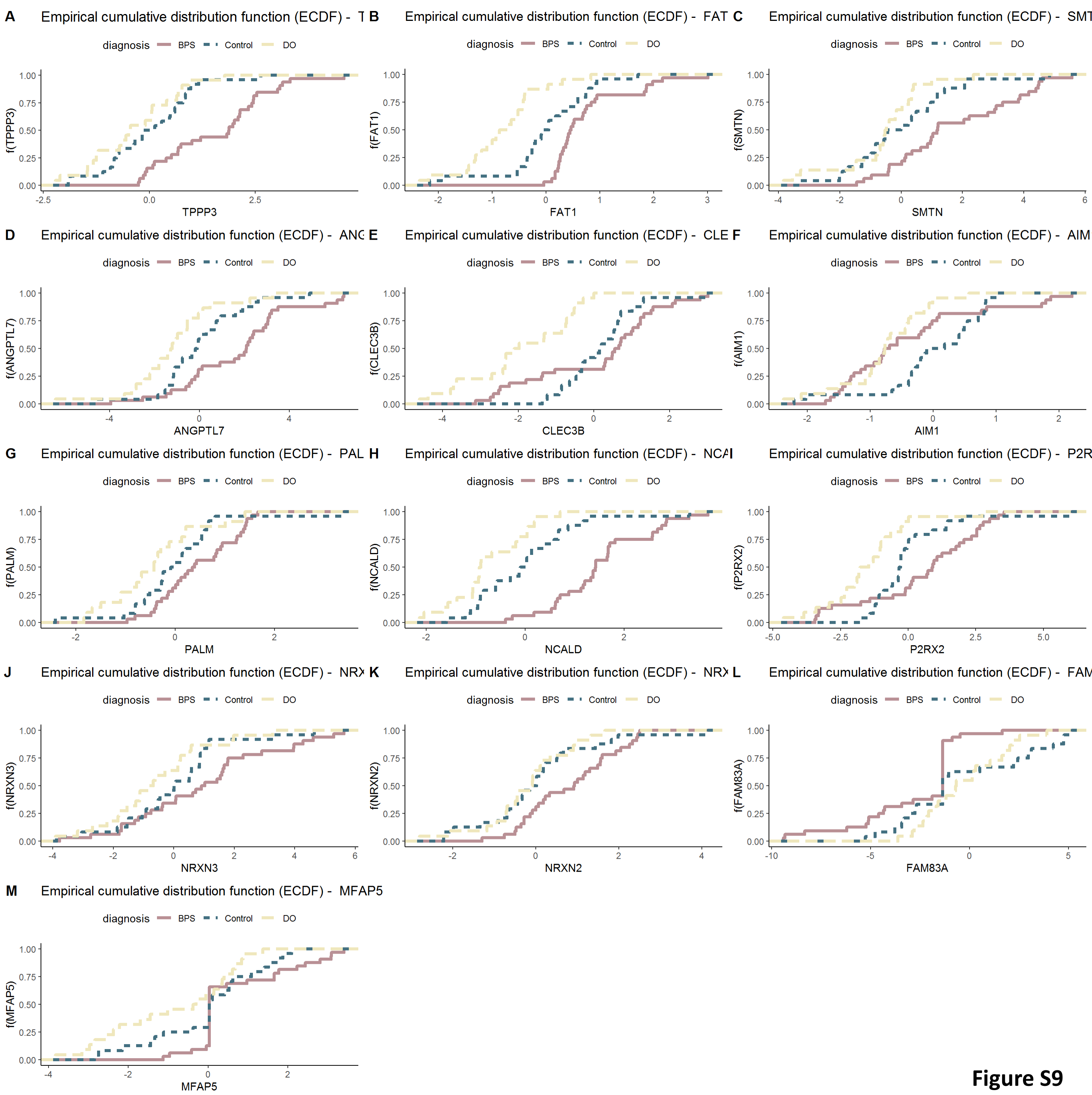
A Empirical cumulative distribution function (ECDF) - T
diagnosis BPS Control DO
f(TPPP3)
TPPP3
B Empirical cumulative distribution function (ECDF) - FAT
diagnosis BPS Control DO
f(FAT1)
FAT1
C Empirical cumulative distribution function (ECDF) - SMT
diagnosis BPS Control DO
f(SMTN)
SMTN
D Empirical cumulative distribution function (ECDF) - ANG
diagnosis BPS Control DO
f(ANGPTL7)
ANGPTL7
E Empirical cumulative distribution function (ECDF) - CLE
diagnosis BPS Control DO
f(CLEC3B)
CLEC3B
F Empirical cumulative distribution function (ECDF) - AIM
diagnosis BPS Control DO
f(AIM1)
AIM1
G Empirical cumulative distribution function (ECDF) - PAL
diagnosis BPS Control DO
f(PALM)
PALM
H Empirical cumulative distribution function (ECDF) - NCA
diagnosis BPS Control DO
f(NCALD)
NCALD
Empirical cumulative distribution function (ECDF) - P2R
diagnosis BPS Control DO
f(P2RX2)
P2RX2
J Empirical cumulative distribution function (ECDF) - NRX
diagnosis BPS Control DO
f(NRXN3)
NRXN3
K Empirical cumulative distribution function (ECDF) - NRX
diagnosis BPS Control DO
f(NRXN2)
NRXN2
L Empirical cumulative distribution function (ECDF) - FAM
diagnosis BPS Control DO
f(FAM83A)
FAM83A
M Empirical cumulative distribution function (ECDF) - MFAP5
diagnosis BPS Control DO
f(MFAP5)
MFAP5

Figure S9

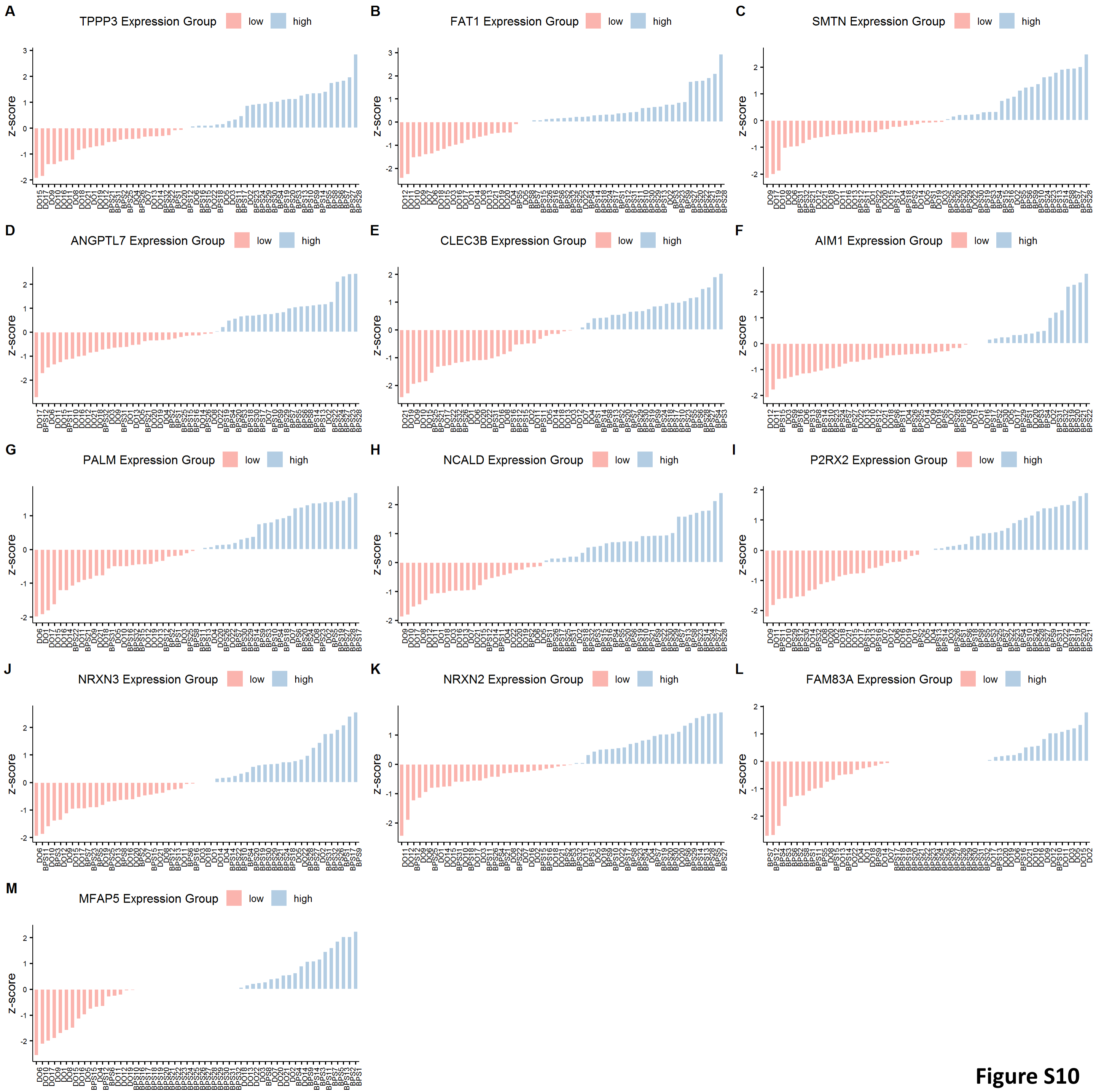
A
TPPP3 Expression Group low high
z-score
B
FAT1 Expression Group low high
z-score
C
SMTN Expression Group low high
z-score
D
ANGPTL7 Expression Group low high
z-score
E
CLEC3B Expression Group low high
z-score
F
AIM1 Expression Group low high
z-score
G
PALM Expression Group low high
z-score
H
NCALD Expression Group low high
z-score
I
P2RX2 Expression Group low high
z-score
J
NRXN3 Expression Group low high
z-score
K
NRXN2 Expression Group low high
z-score
L
FAM83A Expression Group low high
z-score
M
MFAP5 Expression Group low high
z-score

Figure S10

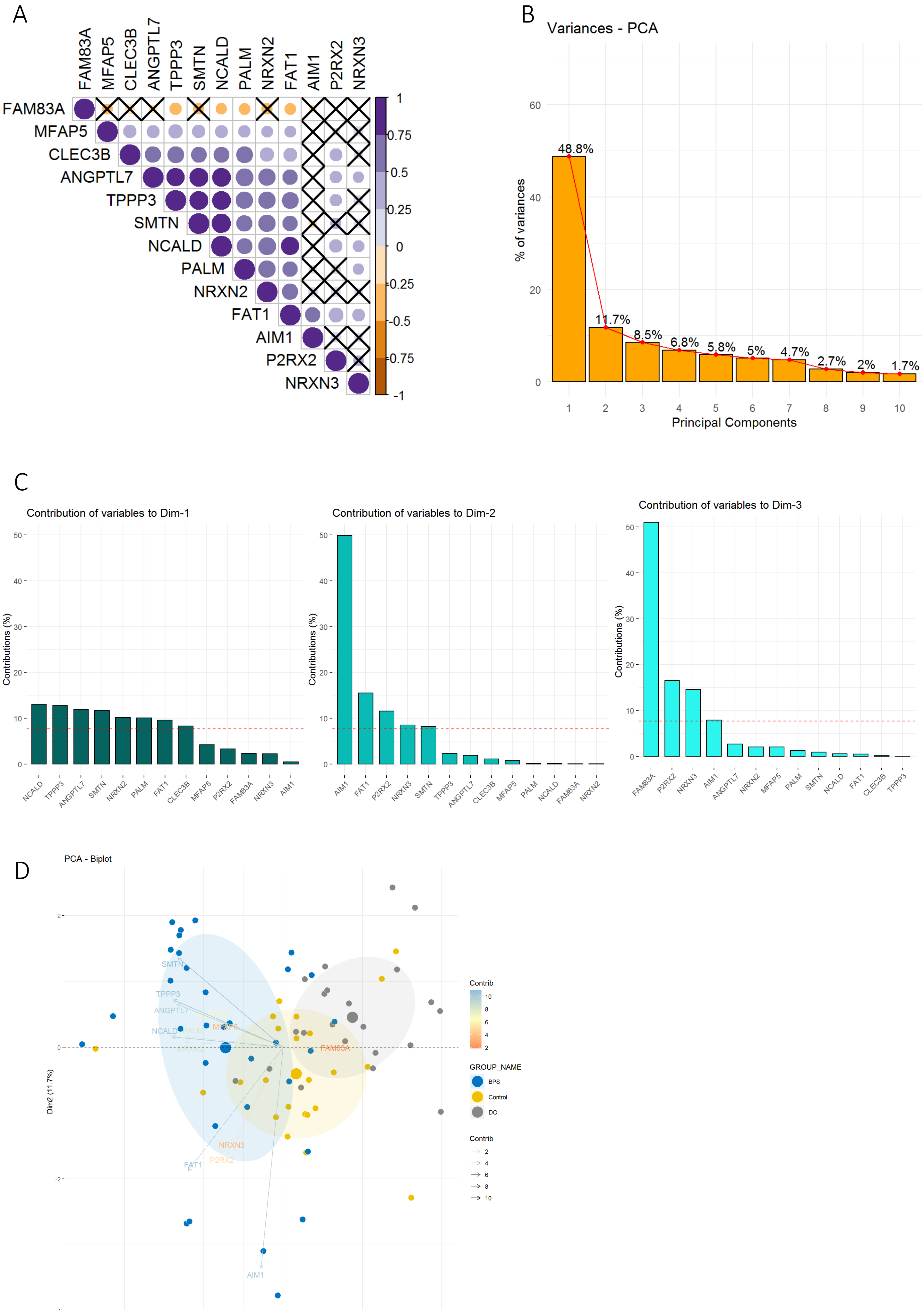
A
FAM83A
MFAP5
CLEC3B
ANGPTL7
TPPP3
SMTN
NCALD
PALM
NRXN2
FAT1
AIM1
P2RX2
NRXN3
B
Variances - PCA
% of variances
Principal Components
48.8%
11.7%
8.5%
6.8%
5.8%
5%
4.7%
2.7%
2%
1.7%
C
Contribution of variables to Dim-1
Contribution of variables to Dim-2
Contribution of variables to Dim-3
Contributions (%)
D
PCA - Biplot
Dim1 (48.8%)
Dim2 (11.7%)
Contrib
GROUP_NAME
BPS
Control
DO

Figure S11

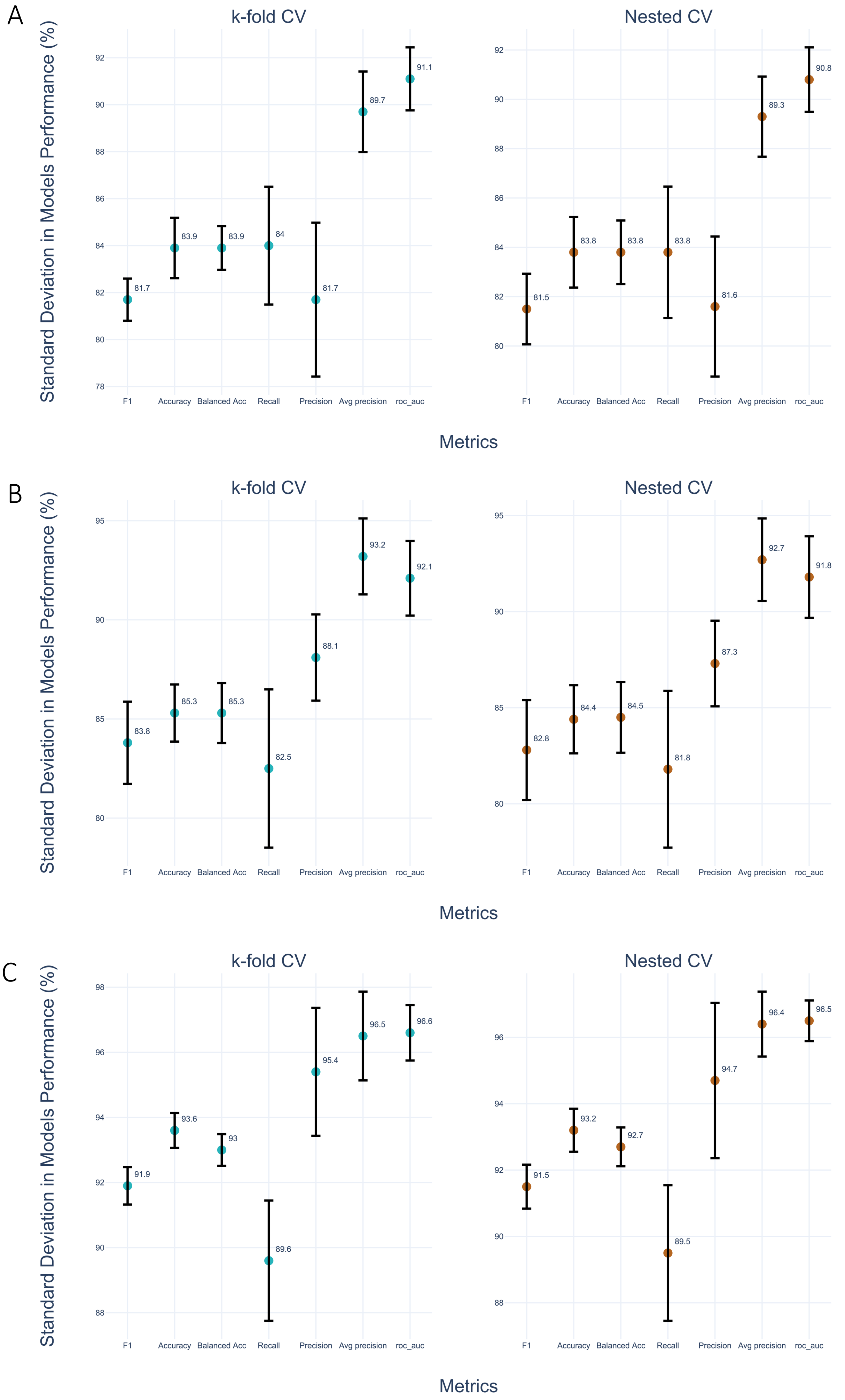
A
k-fold CV
Nested CV
Standard Deviation in Models Performance (%)
F1
Accuracy
Balanced Acc
Recall
Precision
Avg precision
roc_auc
81.7
83.9
83.9
84
81.7
89.7
91.1
81.5
83.8
83.8
83.8
81.6
89.3
90.8
Metrics
B
k-fold CV
Nested CV
Standard Deviation in Models Performance (%)
83.8
85.3
85.3
82.5
88.1
93.2
92.1
82.8
84.4
84.5
81.8
87.3
92.7
91.8
Metrics
C
k-fold CV
Nested CV
Standard Deviation in Models Performance (%)
91.9
93.6
93
89.6
95.4
96.5
96.6
91.5
93.2
92.7
89.5
94.7
96.4
96.5
Metrics

Figure S12

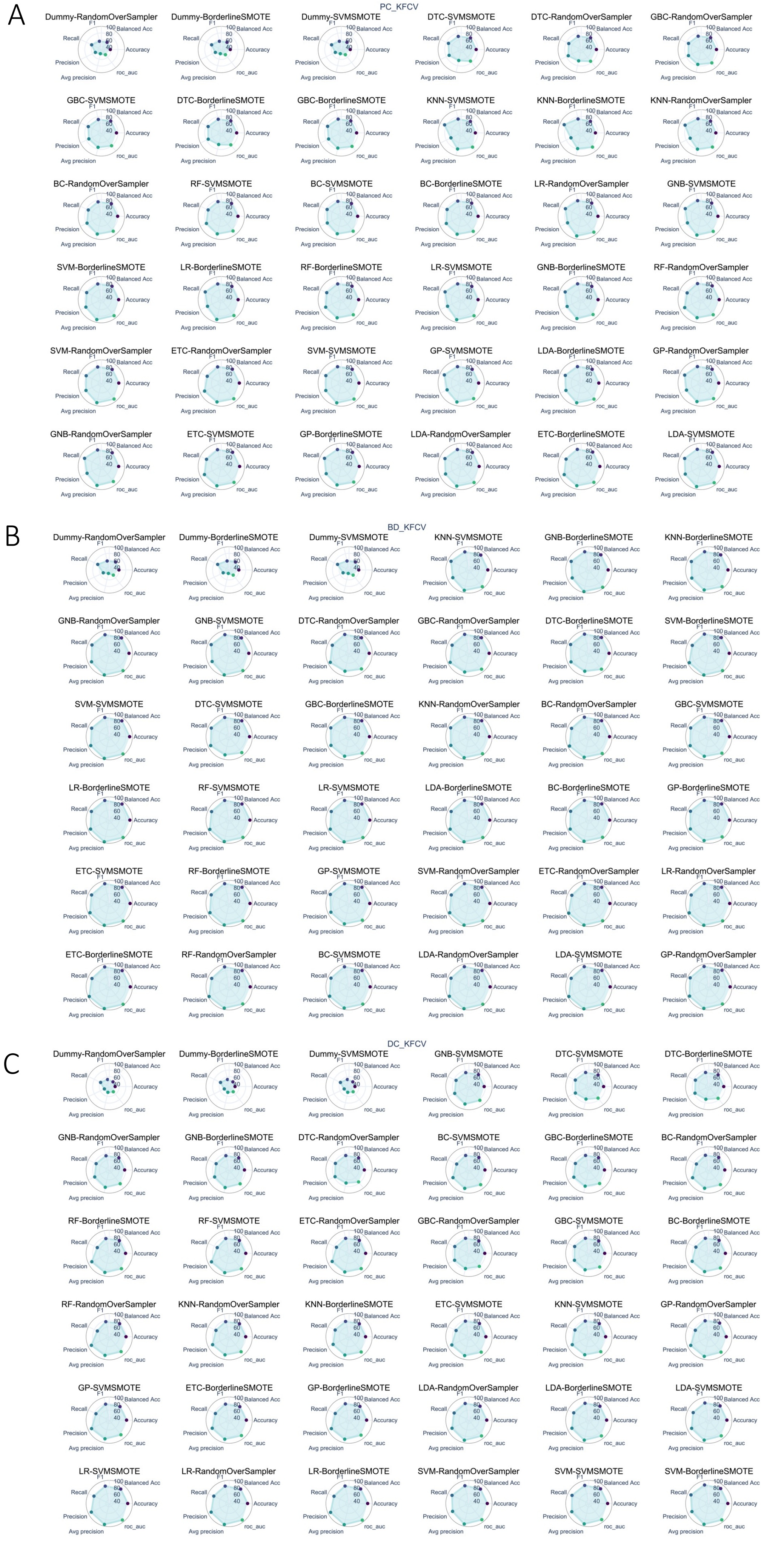
A
PC_KFCV
Dummy-RandomOverSampler
Dummy-BorderlineSMOTE
Dummy-SVMSMOTE
DTC-SVMSMOTE
DTC-RandomOverSampler
GBC-RandomOverSampler
GBC-SVMSMOTE
DTC-BorderlineSMOTE
GBC-BorderlineSMOTE
KNN-SVMSMOTE
KNN-BorderlineSMOTE
KNN-RandomOverSampler
BC-RandomOverSampler
RF-SVMSMOTE
BC-SVMSMOTE
BC-BorderlineSMOTE
LR-RandomOverSampler
GNB-SVMSMOTE
SVM-BorderlineSMOTE
LR-BorderlineSMOTE
RF-BorderlineSMOTE
LR-SVMSMOTE
GNB-BorderlineSMOTE
RF-RandomOverSampler
SVM-RandomOverSampler
ETC-RandomOverSampler
SVM-SVMSMOTE
GP-SVMSMOTE
LDA-BorderlineSMOTE
GP-RandomOverSampler
GNB-RandomOverSampler
ETC-SVMSMOTE
GP-BorderlineSMOTE
LDA-RandomOverSampler
ETC-BorderlineSMOTE
LDA-SVMSMOTE
B
BD_KFCV
Dummy-RandomOverSampler
Dummy-BorderlineSMOTE
Dummy-SVMSMOTE
KNN-SVMSMOTE
GNB-BorderlineSMOTE
KNN-BorderlineSMOTE
GNB-RandomOverSampler
GNB-SVMSMOTE
DTC-RandomOverSampler
GBC-RandomOverSampler
DTC-BorderlineSMOTE
SVM-BorderlineSMOTE
SVM-SVMSMOTE
DTC-SVMSMOTE
GBC-BorderlineSMOTE
KNN-RandomOverSampler
BC-RandomOverSampler
GBC-SVMSMOTE
LR-BorderlineSMOTE
RF-SVMSMOTE
LR-SVMSMOTE
LDA-BorderlineSMOTE
BC-BorderlineSMOTE
GP-BorderlineSMOTE
ETC-SVMSMOTE
RF-BorderlineSMOTE
GP-SVMSMOTE
SVM-RandomOverSampler
ETC-RandomOverSampler
LR-RandomOverSampler
ETC-BorderlineSMOTE
RF-RandomOverSampler
BC-SVMSMOTE
LDA-RandomOverSampler
LDA-SVMSMOTE
GP-RandomOverSampler
C
DC_KFCV
Dummy-RandomOverSampler
Dummy-BorderlineSMOTE
Dummy-SVMSMOTE
GNB-SVMSMOTE
DTC-SVMSMOTE
DTC-BorderlineSMOTE
GNB-RandomOverSampler
GNB-BorderlineSMOTE
DTC-RandomOverSampler
BC-SVMSMOTE
GBC-BorderlineSMOTE
BC-RandomOverSampler
RF-BorderlineSMOTE
RF-SVMSMOTE
ETC-RandomOverSampler
GBC-RandomOverSampler
GBC-SVMSMOTE
BC-BorderlineSMOTE
RF-RandomOverSampler
KNN-RandomOverSampler
KNN-BorderlineSMOTE
ETC-SVMSMOTE
KNN-SVMSMOTE
GP-RandomOverSampler
GP-SVMSMOTE
ETC-BorderlineSMOTE
GP-BorderlineSMOTE
LDA-RandomOverSampler
LDA-BorderlineSMOTE
LDA-SVMSMOTE
LR-SVMSMOTE
LR-RandomOverSampler
LR-BorderlineSMOTE
SVM-RandomOverSampler
SVM-SVMSMOTE
SVM-BorderlineSMOTE
F1
Balanced Acc
Accuracy
roc_auc
Avg precision
Precision
Recall
100
80
60
40

Figure S13

A

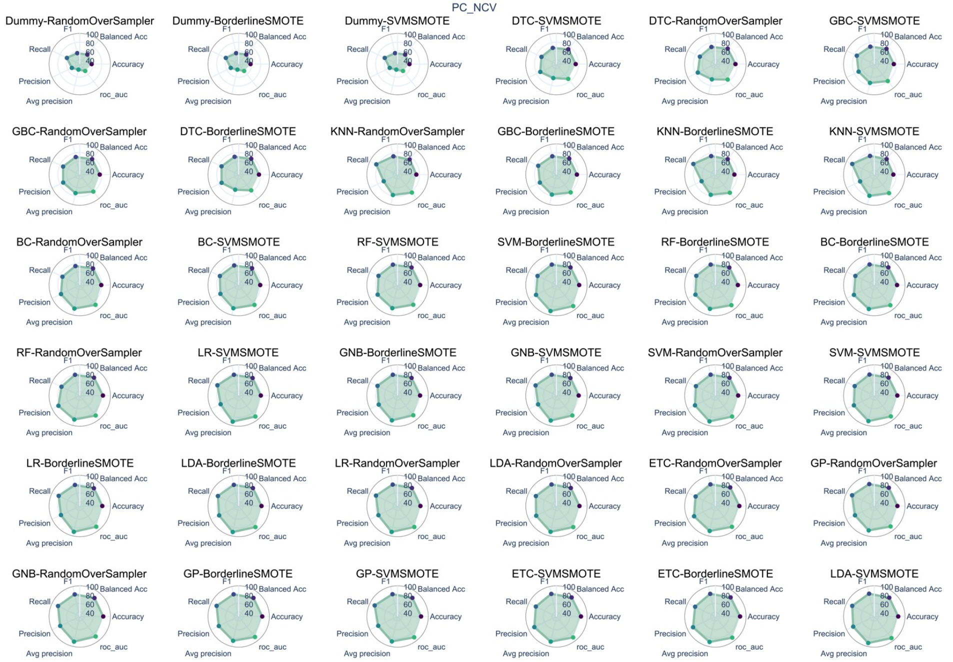

B

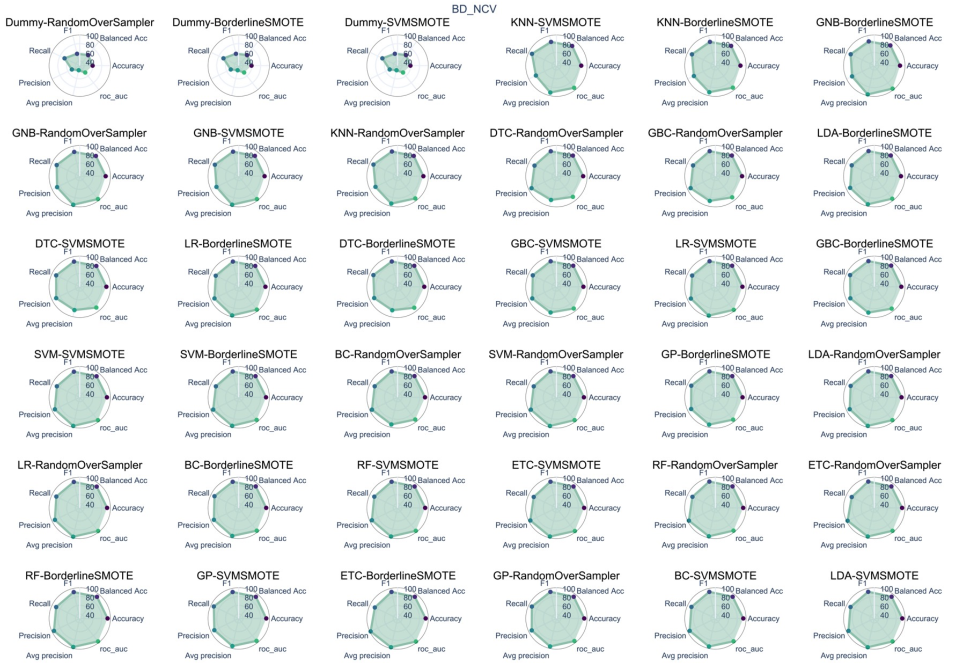

C

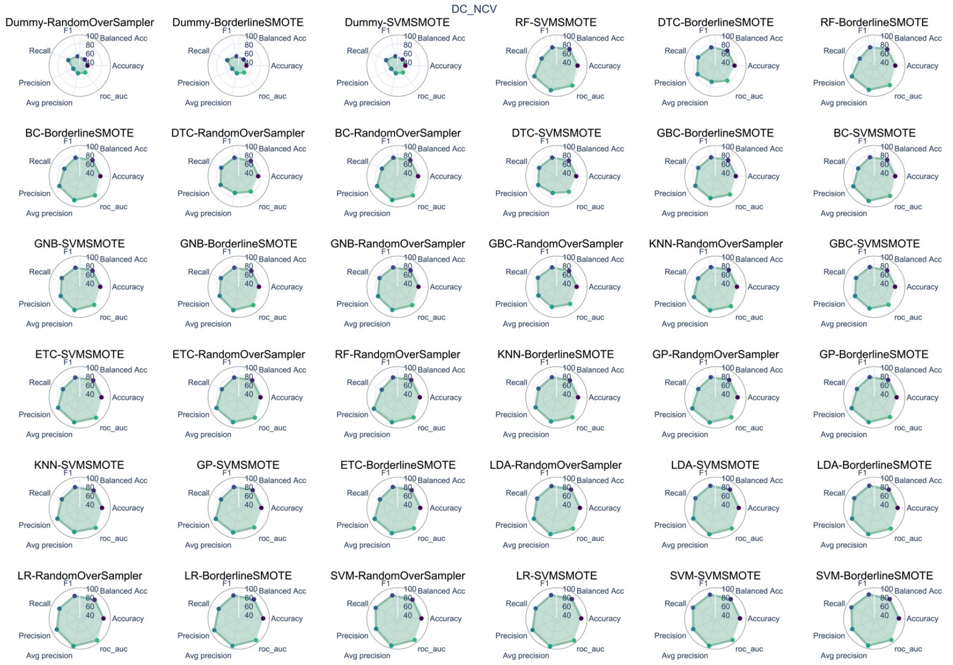

Figure S14

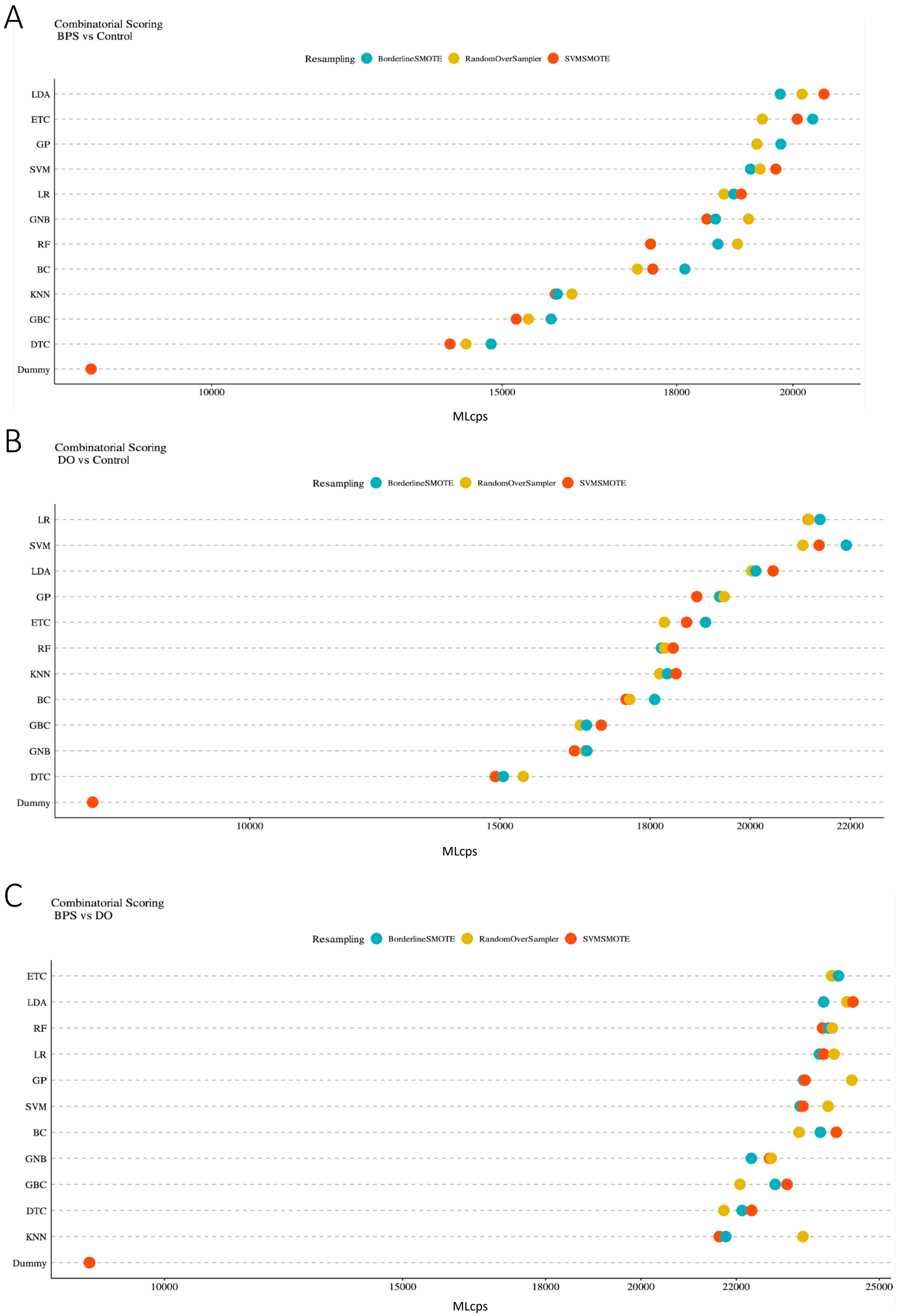

A
Combinatorial Scoring
BPS vs Control
Resampling BorderlineSMOTE RandomOverSampler SVMSMOTE
LDA
ETC
GP
SVM
LR
GNB
RF
BC
KNN
GBC
DTC
Dummy
10000
15000
18000
20000
MLcps
B
Combinatorial Scoring
DO vs Control
Resampling BorderlineSMOTE RandomOverSampler SVMSMOTE
LR
SVM
LDA
GP
ETC
RF
KNN
BC
GBC
GNB
DTC
Dummy
10000
15000
18000
20000
22000
MLcps
C
Combinatorial Scoring
BPS vs DO
Resampling BorderlineSMOTE RandomOverSampler SVMSMOTE
ETC
LDA
RF
LR
GP
SVM
BC
GNB
GBC
DTC
KNN
Dummy
10000
15000
18000
20000
22000
25000
MLcps

Figure S15

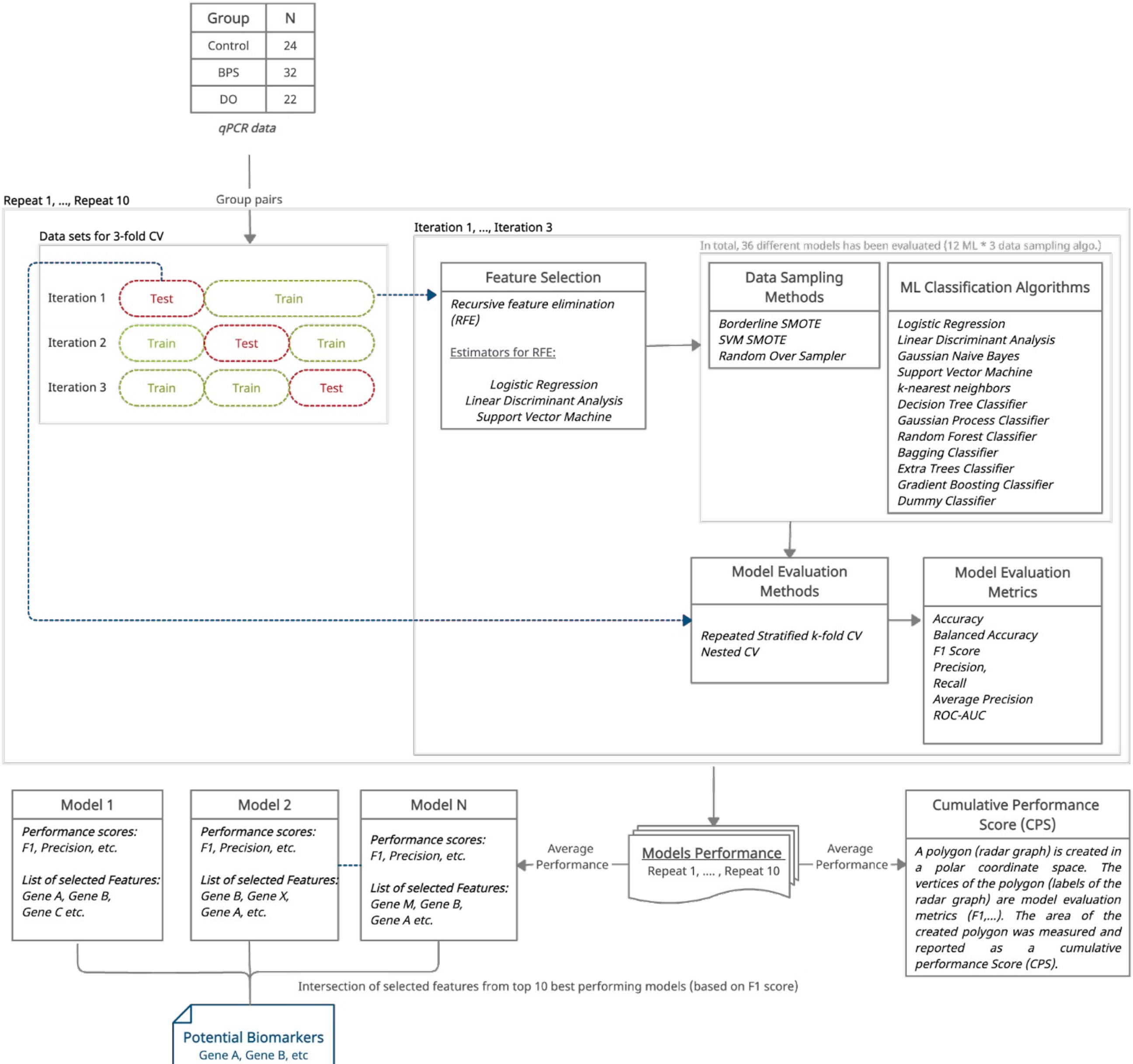

Group
N
Control
24
BPS
32
DO
22
qPCR data
Group pairs
Repeat 1, ..., Repeat 10
Data sets for 3-fold CV
Iteration 1
Test
Train
Iteration 2
Train
Test
Train
Iteration 3
Train
Train
Test
Iteration 1, ..., Iteration 3
In total, 36 different models has been evaluated (12 ML * 3 data sampling algo.)
Feature Selection
Recursive feature elimination (RFE)
Estimators for RFE:
Logistic Regression
Linear Discriminant Analysis
Support Vector Machine
Data Sampling Methods
Borderline SMOTE
SVM SMOTE
Random Over Sampler
ML Classification Algorithms
Logistic Regression
Linear Discriminant Analysis
Gaussian Naive Bayes
Support Vector Machine
k-nearest neighbors
Decision Tree Classifier
Gaussian Process Classifier
Random Forest Classifier
Bagging Classifier
Extra Trees Classifier
Gradient Boosting Classifier
Dummy Classifier
Model Evaluation Methods
Repeated Stratified k-fold CV
Nested CV
Model Evaluation Metrics
Accuracy
Balanced Accuracy
F1 Score
Precision,
Recall
Average Precision
ROC-AUC
Model 1
Performance scores: F1, Precision, etc.
List of selected Features: Gene A, Gene B, Gene C etc.
Model 2
Performance scores: F1, Precision, etc.
List of selected Features: Gene B, Gene X, Gene A, etc.
Model N
Performance scores: F1, Precision, etc.
List of selected Features: Gene M, Gene B, Gene A etc.
Average Performance
Models Performance
Repeat 1, ... , Repeat 10
Average Performance
Cumulative Performance Score (CPS)
A polygon (radar graph) is created in a polar coordinate space. The vertices of the polygon (labels of the radar graph) are model evaluation metrics (F1,...). The area of the created polygon was measured and reported as a cumulative performance Score (CPS).
Intersection of selected features from top 10 best performing models (based on F1 score)
Potential Biomarkers
Gene A, Gene B, etc

Figure S16

Supplementary Figures - Legends

Figure S1. Similarity matrix and normality test of selected genes in NGS dataset. (A) Similarity matrix for DO and BPS patients using 1390 differentially regulated mRNAs in DO and BPS patients purple color show lower similarity and green color represents higher similarity. (B) Normality test for 13 selected markers. For each gene, the normal plots (histogram), Q-Q plot (quantile-quantile plot) of all patients in a single group and Q-Q plot of each patient group separately is calculated.

Figure S2. Functional enrichment analysis of DEGs based on NGS dataset. (A) Enriched GO (biological processes) in differentially expressed genes (DEGs) between DO and control identified using GSEA methods. X-axis indicates LFC values. The color describes the FDR/adjusted p-value as listed in the color bar notifying whether an enriched term is significant (cutoff <0.05). (**B**) Enriched GO (biological processes) in DEGs between BPS and control identified using GSEA methods.

Figure S3. Deviation Graphs of selected 13 markers in NGS datasets of BPS, DO and controls. Deviation Graphs estimating the cumulative distribution of selected genes. Z-score of 1 denotes that the observation is at a distance of one standard deviation to the right from the centre. (A) TPPP3 (B) FAT1 (C) SMTN (D) ANGPTL7 (E) CLEC3B (F) AIM1 (G) PALM (H) NCALD (I) P2RX2 (J) NRXN3 (K) NRXN2 (L) FAM83A (M) MFAP5

Figure S4. Correlation plot and PCA contribution of selected genes in NGS dataset.

(A) Correlation plot visualizing the relationship between the different attributes. Similarity between observations is defined using the inter-observation distance measures or correlation-based distance measures. The relationships with no significance are visualised by a cross on the correlogram. (B) Scree plot of eigenvalues

showing the percentage of variances explained by each principal component. (C) Barplot of gene contributions in the PCA. A reference dashed line is shown on the barplot corresponding to the expected value if the contribution where uniform. For a given dimension, any gene with a contribution above the reference line could be considered as important in contributing to the dimension.

Figure S5. Log2FC distribution of 13 markers in QPCR dataset. Density ridgeline plot of distribution of BPS, DO and controls for a given gene. (A) TPPP3 (B) FAT1 (C) SMTN (D) ANGPTL7 (E) CLEC3B (F) AIM1 (G) PALM (H) NCALD (I) P2RX2 (J) NRXN3 (K) NRXN2 (L) FAM83A (M) MFAP5

Figure S6. Log2FC distribution of the mean of each 13 markers in QPCR dataset. Density ridgeline plot of distribution of BPS and DO and visualizing the mean of each population.

Figure S7. Normality test and PCA contribution of different markers in QPCR dataset. Normality test for 13 selected markers: the normal plots (histogram), Q-Q plot (quantile-quantile plot) of all patients in a single group and Q-Q plot of each patient group separately is calculated.

Figure S8. Testing the performance of selected 13 markers in QPCR datasets of BPS, DO and controls. Boxplot Statistics visualization (Based on log2FC), pairwise comparisons including p-value. (A) TPPP3 (B) FAT1 (C) SMTN (D) ANGPTL7 (E) CLEC3B (F) AIM1 (G) PALM (H) NCALD (I) P2RX2 (J) NRXN3 (K) NRXN2 (L) FAM83A (M) MFAP5

Figure S9. Empirical cumulative distribution function (ECDF) of selected 13 markers in QPCR datasets of BPS, DO and controls. ECDF reports for any given number (mRNA read count) the percentage of individuals that are below that threshold.

(A) TPPP3 (B) FAT1 (C) SMTN (D) ANGPTL7 (E) CLEC3B (F) AIM1 (G) PALM (H) NCALD (I) P2RX2 (J) NRXN3 (K) NRXN2 (L) FAM83A (M) MFAP5

Figure S10. Deviation Graphs of selected 13 markers in QPCR datasets of BPS, DO and controls. Deviation Graphs estimating the cumulative distribution of selected genes. Z-score of 1 denotes that the observation is at a distance of one standard deviation to the right from the centre. (A) TPPP3 (B) FAT1 (C) SMTN (D) ANGPTL7 (E) CLEC3B (F) AIM1 (G) PALM (H) NCALD (I) P2RX2 (J) NRXN3 (K) NRXN2 (L) FAM83A (M) MFAP5

Figure S11. Performance of selected 13 markers (QPCR datasets of BPS, DO and controls) in PCA (A) Correlogram of the relationship between the different attributes. Relationship with no significance is visualised by a cross (B) Scree plot of eigenvalues showing the percentage of variances explained by each principal component. (C) Barplot of gene contributions. For a given dimension, any gene with a contribution above the reference line could be considered as important in contributing to the dimension. (D) PCA biplot showing the separation samples using QPCR data.

Figure S12. Standard deviation of performance metrics for various models. (**A**) Standard deviation of metrics in BPS vs control, (**B**) DO vs control and (**C**) BPS vs DO.

Figure S13. Models' performance based on k-fold cross-validation (CV) method. Each plot represents an ML model, where the first part of the model's name corresponds to a particular ML algorithm, and the latter part indicates a data resampling method. It displays metric scores for the corresponding model, with a higher shaded area indicating better performance. The models are sorted in increasing order based on the F1 score from the k-fold CV result. (**A**) Thirty-six radar graphs illustrate model performances for BPS vs. Control. (**B**) Thirty-six radar graphs illustrate

model performances for BPS vs. DO. (**C**) Thirty-six radar graphs illustrate model performances for DO vs. Control.

Figure S14. Models' performance based on nested cross-validation (CV) method. Each plot represents an ML model, where the first part of the model's name corresponds to a particular ML algorithm, and the latter part indicates a data resampling method. It displays metric scores for the corresponding model, with a higher shaded area indicating better performance. The models are sorted in increasing order based on the F1 score from the k-fold CV result. (**A**) Thirty-six radar graphs illustrate model performances for BPS vs. Control. (**B**) Thirty-six radar graphs illustrate model performances for BPS vs. DO. (**C**) Thirty-six radar graphs illustrate model performances for DO vs. Control.

Figure S15. MLcps Results. MLcps scores are depicted in Cleveland's dot plot for (**A**) BPS vs. control, (**B**) DO vs. control, and (**C**) BPS vs. DO.

Figure S16. Proposed ML Framework. The proposed pipeline first splits the datasets into k (3) equal-sized bins in a stratified manner, where k-1 bins will be used as training datasets and the remaining bin as a test dataset. Next, it employs the RFECV method for feature selection, three data resampling techniques, and twelve ML algorithms. Then, it evaluates the model performance on the test dataset using the k-fold and nested CV (k=3) method and calculates seven different performance metrics. As part of the k-fold CV methods, it repeats the last two steps for each unique bin. It repeats the complete process ten times and takes the average performance as the final model performance. In the end, the pipeline provides a list of the intersection of selected features from the top 10 best-performing models (based on F1 score) as the final list of selected features.
